# Determinants of welfare benefit use of immigrant groups - longitudinal evidence from Germany

**DOI:** 10.3389/fsoc.2022.839352

**Published:** 2022-11-04

**Authors:** Emily Frank

**Affiliations:** ^1^Berlin Institute for Integration and Migration Research (BIM), Berlin, Germany; ^2^Department of Governance, Hertie School, Berlin, Germany; ^3^Department of Social Sciences, Humboldt University of Berlin, Berlin, Germany

**Keywords:** migration, refugees and asylum seekers, labor market, human capital, immigrant integration, economics of immigration, legal status, immigration labor policy

## Abstract

While recent literature in Germany has compared predictors of welfare use between EU and non-EU immigrants, refugees have yet to be added to the analysis. Using survey data of approximately 4,000 immigrants living in Germany, I examine the determinants of basic unemployment benefits receipt for intra-EU immigrants, refugees, and third country immigrants. In particular, I investigate how education affects the likelihood of welfare use for each immigrant group. Even after controlling for human capital factors, sociodemographic characteristics, and factors related to migration such as legal status and age at migration, refugees remain significantly more likely to receive benefits. Results demonstrate that higher education significantly decreases the likelihood of welfare receipt for EU and third country immigrants, but much less so for refugees. These findings may indicate that refugees' education is not being used to its full potential in the labor market or that they face additional challenges hindering their labor market integration. A further and unanticipated finding is that immigrants who hold permanent residency or German citizenship are less likely to receive unemployment benefits, pointing either to positive effects of a secure residency or selection into permanent residency and citizenship among those with the greatest labor market success. Overall, this research shows that challenges beyond human capital deficiencies and sociodemographic characteristics must be considered when studying immigrants' receipt of social benefits, that not all educational credentials are valued equally, and that the experiences of refugees differ in significant ways from those of other immigrant groups.

## 1. Introduction

Nearly 13% of Germany's population, or just over 10 million people, are first-generation immigrants (BAMF/BMI, 2019). Many immigrants moved to Germany in the 1950s and 1960s as part of “guest worker” programs, which recruited workers from a number of countries, primarily Turkey as well as several southern European countries, North African countries, and Yugoslavia. A large number of ethnic Germans also moved to Germany in the 1980s and 1990s, mainly from the Soviet Union, Poland, and Romania. Finally, nearly 2 million people applied for asylum in Germany between 2013 and 2018, more than 30% of all asylum applications in the EU, with the largest group arriving from Syria due to civil conflict (BAMF/BMI, 2019). Since the 2015 “summer of migration,” Germany has emerged as a leading destination for migration to Europe.

Despite Germany's diverse immigrant populations, there has been little research investigating their use of welfare benefits. For the most part, the existing literature has focused on comparisons between immigrants and natives. These studies largely indicate that immigrants depend on welfare more than natives due to sociodemographic factors, including single parenthood and a larger number of children (Riphahn, 1998; Castronova et al., 2001; Barrett and Maître, 2013; Bruckmeier and Wiemers, 2017). What has been somewhat overlooked is the gap in unemployment benefit receipt between immigrant groups. Wunder and Riphahn (2014) conducted one of the few studies comparing welfare benefit receipt between EU and non-EU immigrants in Germany. They identify patterns of higher welfare persistence among non-EU citizens compared to EU citizens. Although they attribute some of this pattern to differences in human capital and sociodemographic characteristics, non-EU immigrants exhibit a higher rate of welfare dependence even after accounting for these factors (Wunder and Riphahn, 2014). Furthermore, Wunder and Riphahn carried out their study before the 2015–2016 refugee “crisis” and thus do not draw conclusions about asylum seekers, now one of the largest immigrant groups in Germany. To date, the welfare receipt of different immigrant groups has not been investigated further.

The welfare use of refugees merits further investigation and comparison with the other two immigrant groups. Building on the work of Wunder and Riphahn (2014), I compare predictors of unemployment benefits receipt between EU and non-EU citizens living in Germany, adding refugees to the analysis as a separate category. Using the German Socio-Economic Panel (SOEP), a large-scale longitudinal survey, I analyze data on over 4,000 immigrants surveyed from the years 2013 to 2019 and examine predictors of welfare receipt for EU, non-EU and refugee immigrants in Germany. I include human capital and sociodemographic factors already shown to be significant in the existing literature as well as legal status, age at immigration, and years of residency in Germany - all important factors related to immigrants' labor market trajectories.

I consider especially the role of immigrants' human capital in predicting welfare receipt, and whether this relationship is affected by immigrants' countries of origin and reasons for moving to Germany. Human capital theory predicts that greater education, work experience, and language skills should contribute to increased labor market success, leading to a lower likelihood of welfare receipt. However, immigrants' human capital may be valued differently in the German labor market depending on where it was acquired. Education and qualifications from one country may not lead to the same chances of employment as education and qualifications from another country. While Wunder and Riphahn's results demonstrate that education is a strong predictor of systematic differences in welfare receipt between EU and non-EU immigrants, they do not compare the likelihood of welfare receipt between immigrants with the same education level. Extensive research in Western countries has indicated that immigrants from other Western countries enjoy greater labor market returns to education than those from non-Western countries (Basilio et al., 2017; Lancee and Bol, 2017), while education acquired in the destination country leads to the highest returns (Schoeni, 1997; Friedberg, 2000; Bratsberg et al., 2002).

This article makes several important contributions to the literature on immigrant labor market integration. To the best of my knowledge, this is one of the first studies since the recent wave of refugees to compare human capital determinants of welfare use between immigrant groups in Germany. I provide evidence concerning differences in returns to education between EU immigrants, refugees, and other third-country immigrants, supporting labor market integration policies directed at immigrants and refugees. The effects of education on likelihood of welfare receipt are comparable for EU and third country immigrants, but are relatively minimal for refugees. Yet even after accounting for human capital, sociodemographic factors, and several factors related to respondents' immigration pathways, EU immigrants are the least likely to receive welfare benefits and refugees remain the most likely. Such systematic differences between immigrant groups may occur due to the devaluation of educational qualifications from certain countries, differences between sending countries' education systems and labor markets, or discrimination. Finally, my results demonstrate that permanent residency or citizenship may potentially support positive labor market outcomes across immigrant groups.

## 2. Theory and hypotheses

### 2.1. Predictors of welfare receipt

Hohmeyer and Lietzmann (2020) attribute the likelihood of welfare receipt to two mechanisms. For one matter, longer duration of welfare receipt can increase the likelihood of future benefit receipt. This is because welfare receipt decreases employment chances, both by sending a negative signal to potential employers (Blank, 1989; Lockwood, 1991) and by leading to human capital deterioration during extended periods of inactivity (Gregory and Jukes, 2001). Negative consequences for mental health and motivation can also contribute to a decrease in employment chances (Jahoda, 1982).

In addition, individuals with good prospects of employment are less likely to receive benefits. Many factors contribute to employment prospects, including job skills, the availability of employment support systems such as transportation and childcare, and mental health factors (Blumenberg, 2002). Finally, one major predictor of employment prospects is human capital (Mincer, 1958, 1991; Nickell, 1979). Human capital theory anticipates better labor market outcomes among those possessing greater skills (Becker, 1964). Primarily through education, individuals increase their skills and therefore their likelihood of employment and higher wages (Nickell, 1979; Mincer, 1991; Cairó and Cajner, 2018). Other forms of human capital include work experience (Mincer and Polachek, 1974) and host-country language skills (Chiswick and Miller, 2003).

### 2.2. Immigrants' human capital

Immigrants in Germany display heterogeneity in terms of human capital. While education levels are rising across countries of origin, immigrants from the EU tend to demonstrate higher education levels than other immigrants (Kogan, 2011; Gries et al., 2021). Conversely, immigrants from non-Western countries tend to demonstrate the greatest educational disadvantages (Brücker et al., 2016a; Spörlein et al., 2020). These differences may be due in part to education systems in immigrant-sending countries. Not all immigrant-sending countries rely on professional training and educational qualifications to the same degree as Germany (Souto-Otero and Villalba-Garcia, 2015). In many third countries, formal vocational training is much less common than in Germany and much fewer occupations require formalized training (Stoewe, 2017). For this reason, certain countries may be more likely to send more skilled immigrants than others because of overall education levels in the country.

Selection mechanisms also contribute to systematic variation in human capital between immigrants in different visa categories (Aydemir, 2011). Immigrants who migrate for work purposes are positively selected based on the skills and qualifications desired by employers (Kontos, 2011; Boeri et al., 2012). As such, immigration processes are easier for those possessing certain qualifications and skills, and there are even particular residence permits to enable highly skilled workers to more easily migrate to Germany (Cerna, 2013; Ellermann, 2020). Comparatively, refugees are less positively selected on human capital as they migrate due to humanitarian reasons (Chiswick, 1999; Dustmann et al., 2017; Brell et al., 2020). They therefore face labor market disadvantages compared to economic immigrants (Aydemir, 2011; Bevelander, 2011; Salikutluk et al., 2016). This may be one explanation as to why refugees in Germany demonstrate lower average education levels compared to other immigrants (Brücker et al., 2016a).

### 2.3. Devaluation of origin-country human capital

Although substantial literature confirms that human capital is a significant predictor of welfare receipt, there is also a large body of research indicating differences in returns to human capital for immigrants from different countries of origin. A key question is therefore whether human capital predicts welfare receipt to the same extent for all immigrants. There are several reasons why immigrants' human capital assets may bring them limited employment returns, including devaluation of origin-country human capital, skill mismatches, discrimination, and return migration.

The human capital literature distinguishes between general human capital and country-specific human capital (Chiswick, 1978; Borjas, 1985; Friedberg, 2000). Significant research has established that origin-country human capital is not valued in the labor market as much as destination-country human capital (Schoeni, 1997; Friedberg, 2000; Bratsberg et al., 2002). Furthermore, immigrants' origin country matters: non-Western degrees or degrees from countries with lower levels of economic development are associated with lower wages compared to degrees from Western countries (Lancee and Bol, 2017). Friedberg (2000) finds that returns to education are higher for immigrants from Europe and the Western Hemisphere than for immigrants from Asia and Africa. A recent analysis from Basilio et al. (2017) indicates that the native-immigrant earnings gap in Germany can be mostly explained by heterogeneity in returns to human capital by country, with higher returns for immigrants from high-income countries.

Origin-country human capital is devalued both in formal and informal ways. For one matter, foreign degrees must be recognized by the government's Central Office for Foreign Education in order to enter many professions. Degrees are recognized based on evaluations of quality and comparability to the German education system. Non-Western educational and professional qualifications are less likely to be granted recognition (Bauder, 2005; Brussig et al., 2013; Damelang and Abraham, 2016). Furthermore, legal mechanisms such as the EU Recognition Directive (2005) institute recognition of qualifications from other EU member states, the European Economic Area (EEA), and Switzerland. Immigrants from non-Western and non-European countries may therefore suffer in the labor market due to lack of formal recognition of their qualifications. Evidence indicates that degree recognition results in positive labor market outcomes in terms of employment and earnings (Brücker et al., 2021).

In addition to formal recognition, educational credentials from different countries may be valued differently by potential employers. These valuations can be based on actual or perceived quality of credentials. Immigrants from developed countries, which can devote more government resources to education, may enjoy a better quality of home country education, leading to greater cognitive skills and consequently more job market success (Altinok et al., 2018). However, Lancee & Bol provide an alternate explanation, suggesting that employers perceive non-Western degrees and credentials to be of lower value, regardless of their actual quality. They refer to signaling theory to explain this valuation process. As employers do not have complete information about job seekers' skills and productivity, they use degrees and credentials as signals or cues on which to base hiring decisions (Spence, 1973; Weiss, 1983). In this manner, employers' notions of skill and qualification replicate historical inequalities between Western and non-Western countries (Nowicka, 2014). Lancee and Bol find that the lower wages of non-Western workers can be explained at least in part by the lower signaling value of non-Western degrees. Lack of formal and informal recognition of educational credentials could lead to challenges finding employment or earning a sufficient income, increasing likelihood of welfare receipt. Because of these differences in the transferability of qualifications, I hypothesize *a greater negative effect of educational attainment on welfare receipt for EU immigrants than for third country immigrants and refugees* (H1).

### 2.4. Other potential explanations

Immigrants experience not only devaluation of education from the country of origin, but also additional labor market disadvantages that limit their returns to education. These factors may contribute to a gap in welfare receipt between immigrant groups left unexplained by human capital and sociodemographic factors. For one matter, research on labor market discrimination shows that third country immigrants experience greater discrimination based on ethnicity compared to EU immigrants (Rydgren, 2004; Constant and Massey, 2005; Kaas and Manger, 2012; Thijssen et al., 2021).

Differences between origin-country and host-country education systems and labor markets can lead to skill mismatches. Some studies have indicated that labor market returns to human capital are positively related to the sending country's GDP (Sullivan, 2010; Lagakos et al., 2012; Coulombe et al., 2014). Migrants from countries with labor markets similar to that of Germany potentially fit better into the German labor market in terms of jobs and skills needed (Duleep and Regets, 1999). Migrants from OECD and EU countries may also simply be more familiar with the German labor market system due to similarities with the origin country (Van Tubergen et al., 2004; Beyer, 2017).

Finally, immigrants who are unsuccessful in the labor market may be more likely to return to the country of origin. However, evidence regarding the link between unemployment and return migration is mixed. Several studies have indicated that unemployment spells increase return probability across immigrant groups (Constant and Massey, 2002; Gundel and Peters, 2008; Bijwaard et al., 2014). Others find that social networks, perceived discrimination, and calculations of wage differentials between the origin and destination countries can all contribute to return migration decisions (Borjas and Bratsberg, 1994; Waldorf, 1995; Tezcan, 2019). Freedom of movement within the European Union may enable EU immigrants to return home more easily if they fall upon hard times (Zaiceva and Zimmermann, 2016). By contrast refugees are unlikely to be able to quickly return to their country of origin due to ongoing violence and conflict.

All of the above factors can lead to differences in labor market success even between immigrants from different sending countries but with the same educational qualifications. A large body of evidence indicates that immigrants with credentials from non-Western countries experience lower labor market returns to their education than those from Western or OECD countries. These decreased labor market returns lead to higher likelihood of welfare receipt. Other factors including skill mismatches, return migration, discrimination, and age at migration. Because of these differences, I hypothesize that *EU immigrants exhibit the lowest rate of welfare use compared to third country immigrants and refugees (H2a)*.

### 2.5. The refugee penalty

Building on previous research comparing the welfare use of immigrant groups, this paper adds refugees to the analysis as a separate group from other third country immigrants. Studies conducted after the arrival of recent refugees have indicated a refugee labor market penalty that cannot be completely explained by differences in education level or sociodemographic factors (Bähr et al., 2017; Söhn, 2019; Bedaso, 2021). However, comparison between recent refugees and other immigrants has been limited. Because of their additional disadvantages, I hypothesize an *additional refugee penalty predicting higher use of welfare benefits, even after accounting for human capital and sociodemographic factors (H2b)*.

There are several potential explanations for the “refugee penalty.” For one matter, refugees face labor market discrimination. Recent studies have indicated that even well-qualified refugees who have learned German encounter challenges finding work and are evaluated not only based on their skills, but also on their country of origin. Employers frequently assume that refugees are less capable than other equally-educated immigrants (Khan-Gökkaya and Mösko, 2021; Kloubert and Hoggan, 2021). In addition, mental health issues stemming from traumatic events may hinder the labor market integration of refugees (Ruiz and Vargas-Silva, 2018; Brell et al., 2020). Legal regulations governing residence and employment can also negatively affect refugees' employment prospects. Employers may be met with high costs if they choose to hire refugees who have only received a delay of deportation - rather than protection status - and cannot be certain that refugees without protection status will remain in Germany (Brücker et al., 2016b). In addition, employment prohibitions upon arrival in Germany may discourage asylum applicants from seeking work later once permission is eventually granted (Brell et al., 2020; Fasani et al., 2021). The refugee penalty merits further exploration to determine how these factors may affect labor market outcomes.

### 2.6. Germany's welfare regime

Two types of welfare benefits are available in Germany for unemployed adults of working age: Unemployment Benefits I (UB I) and Unemployment Benefits II (UB II). Eligibility for UB I is based on social security contributions. Recently unemployed workers who have contributed sufficiently to the welfare system receive a benefit amount calculated from previous earnings. Those who have exhausted their UB I benefit or who have not worked long enough to qualify for UB I can receive UB II. Rather than social security contributions, UB II is a means-tested benefit, meaning that eligibility is based on income and need. The benefit amount is set according to the number of people in the household, the number of children in the household, and the ages of the children. In general, all residents of Germany are entitled to UB II, excluding tourists, seasonal workers, and asylum seekers. Recipients are also required to be between the ages of 15 and 65 and be able to work at least 15 h per week.

UB II receipt is conditional on receiving sufficient income to secure a minimum standard of living, but not conditional on employment. Individuals may be registered as employed but not earn enough to meet this minimum standard, and can therefore receive UB II to fill the gap. Conversely, unemployed individuals may not necessarily receive UB II if they have not applied or if the application process is incomplete. The relationship between unemployment and UB II receipt is therefore not always direct (Hohmeyer and Lietzmann, 2020).

EU citizens are broadly entitled to UB II in Germany under the Freedom of Movement Law (FreizügG/EU), a German national law derived from EU-level directives. There are some conditions upon recipiency: EU citizens cannot claim social security benefits for their first 3 months in Germany, as they are not permitted to enter Germany with the purpose of claiming social benefits (Mantu and Minderhoud, 2017). To become eligible, they must either work and contribute to the social security system for at least 1 year or reside in Germany for at least 5 years. As of 2017, economically inactive EU citizens cannot receive any type of welfare benefit during their first 5 years in Germany (see *Bundesgesetzblatt* Nr. 65, *Bundesregierung* 12/28/2016). If they have worked for less than 1 year, they are entitled to a maximum of 6 months of UB II benefits (see *Bundesgesetzblatt* Nr. 65, *Bundesregierung* 12/28/2016).

Migrants from several countries outside of the EU are granted special access to the welfare system under the European Convention on Social and Medical Assistance of 1953. These countries include Iceland, Norway, and Turkey. Other third country immigrants living in Germany can be eligible if they have a residence permit and legal permission to work. However, UB II receipt can increase the risk rejection or delay of residency permit extensions or German citizenship applications (Schnabel, 2020).

Refugees who have officially received legal protection status fall under largely the same eligibility requirements for UB II as German citizens (see *Sozialgesetzbuch II - Grundsicherung für Arbeitsuchende* § 7 *Leistungsberechtigte, Bundesregierung* 12/24/2003). Finally, refugees waiting for an asylum decision (or those who possess a *Duldung*, or temporary suspension of deportation) are entitled to an asylum seekers' benefit (*Asylbewerberleistungsgesetz*, AsylbLG), rather than UB II. The above information on immigrants' eligibility for UB II is also available in the Supplementary Table S1.

## 3. Data and methodology

### 3.1. Data

Data for my analyses are taken from the 2013 to 2019 waves of the core sample of the German Socioeconomic Panel (SOEP), a longitudinal panel dataset which includes representative samples of the German population (Socio-Economic Panel (SOEP), 2021). Running since 1984, the SOEP re-interviews adult household members annually and has frequently added new samples for the purpose of studying various social groups. My analytical sample includes all UB II-eligible heads of household with a direct migration background who were surveyed in the years 2013–2019. Some respondents have participated in the SOEP since its commencement, such as those from “Sample B Foreigners in the Federal Republic of Germany,” which began in 1984 and includes individuals in private households with a Turkish, Greek, Yugoslavian, Spanish, or Italian household head. Others entered the survey later, including “Sample D Immigrants,” begun in 1994. In 2013 and 2015, two new Migration Samples were incorporated, one including those who immigrated to Germany after 1995 and the second including those who immigrated between 2009 and 2013 (IAB-SOEP Migration Samples, 2021). Refugee Samples were added in 2016 and 2017 and surveyed individuals who arrived in Germany between January 2013 and December 2016 with an asylum application (IAB-BAMF-SOEP Survey of Refugees, 2021). The analytical sample therefore includes individuals who have resided in Germany from less than 1 year up to more than 50 years.

Based on eligibility for UB II, I restrict the sample as follows: first, I include only first-generation immigrant household heads surveyed from the years 2013 to 2019. The sample is further restricted to those between the ages of 18 and 65^1^ who are not on parental leave and not receiving old-age, disability or civil service benefits, as receipt of these benefits precludes UB II receipt. The sample is divided into three immigrant groups: those who migrated from EU countries, those who migrated from third countries, and those who arrived as refugees. Further detail regarding excluded cases is available in the Supplementary Table S2.

As legal status shapes eligibility for social benefits in Germany (Voigt, 2020), this is also a necessary sampling criterion. Because EU citizens only become eligible for UB II after either working for at least 1 year or residing in Germany for 5 years, I exclude EU citizens who have lived in Germany for less than 5 years and do not have at least 1 year of full-time work experience^2^. Asylum seekers are excluded from the sample, as they are not eligible for UB II and receive asylum seekers' benefits instead. Asylum recipients are included as they are entitled to UB II.

Finally, the analytical sample includes only immigrants whose highest educational qualifications were acquired abroad. As noted above, when immigrants receive their education in the destination country, they typically enjoy greater labor market returns than those educated in the origin country. Educational qualifications from Germany are therefore not comparable to those from abroad, and those immigrants who received their highest qualification from Germany are excluded.

### 3.2. Measurement

The dependent variable in the analysis is UB II receipt. UB II receipt is measured at the household level, with a binary indicator of 1 for benefit receipt and 0 for no benefit receipt. Like most of the variables in the SOEP, this variable is self-reported, based on questions asking households which benefits they are receiving and the amount that they receive. The independent variable and control variables are operationalized dependent on how survey questions are asked in the SOEP. Education level serves as the independent variable. Control variables for the various stepwise models include: unemployment experience; human capital factors including full-time employment experience and German language skills; sociodemographic factors including gender, number of children in the household, and single parenthood; and factors related to immigration including legal status, age at immigration, and length of time the respondent has resided in Germany^3^. Although I provide further details regarding these variables below, an additional table explaining the precise operationalization of model components is also available in the Supplementary Table S4.

The first variables to be added in the stepwise modeling process are the human capital variables. Education level is measured through the International Standard Classification of Education (ISCED), which provides a standardized cross-national qualification of educational degrees to capture education obtained prior to arrival in Germany^4^. This measure is then simplified into three categories for analysis: basic education, which includes primary and lower secondary education; medium-level education, which includes upper secondary, post-secondary non-tertiary, and short-cycle tertiary education; and higher education, which includes bachelor's, master's, and doctoral degrees.

I also include work experience as a form of human capital. The “full-time employment experience” variable indicates the total length of full-time employment in the respondent's career up to the point of the interview. As Hohmeyer and Lietzmann (2020) attribute likelihood of welfare receipt to employment prospects as well as duration of welfare receipt to date, I also include length of time unemployed. The “unemployment experience” variable indicates the total length of unemployment in the respondent's career and is included to reflect Hohmeyer and Lietzmann's observation of the role of previous welfare receipt in predicting ongoing welfare receipt. Both variables measure length of time in years.

German language ability is a final component of country-specific human capital. I compile this variable from several similar questions about oral or spoken language abilities, all of which asked respondents to rate their language abilities on a scale of 1–5, with 5 being the highest ability; for some respondents surveyed in 2018, this question was not asked, and so I imputed language proficiency from 2017 based on the assumption that their oral German abilities would remain at least at the level of the previous year.

In addition to human capital factors, sociodemographic factors are added in the stepwise modeling process. A number of studies in the German context have indicated that a higher welfare participation rate among immigrants compared to natives is in large part due to sociodemographic factors (Riphahn, 1998; Castronova et al., 2001; Barrett and Maître, 2013; Bruckmeier and Wiemers, 2017). These factors also contribute to higher welfare receipt among non-EU immigrants compared to EU immigrants (Wunder and Riphahn, 2014). More specifically, single parenthood, a female household head, and a larger number of children increase risk of welfare dependence (Riphahn, 1998; Fertig and Schmidt, 2001; Anastossova and Paligorova, 2006). Considering this body of literature, I control for gender, single parenthood, and number of children in the household. The variable indicating the number of children is a categorical variable, with three categories: no children, one child, or multiple children.

Finally, several variables related to respondents' immigration trajectories are included in the models: legal status, age at immigration, and years of residence in Germany. Immigrants who arrive in the destination country at a younger age demonstrate labor market advantages for several reasons, including greater opportunities to gain language fluency, assimilate into the culture, join the education system and invest in other country-specific human capital (Gustafsson et al., 2017; Friedberg, 1992). Several studies indicate that immigrants who arrive in the destination country at a younger age display better labor market outcomes in adulthood (Van den Berg et al., 2014; Lemmermann and Riphahn, 2018). Age at immigration is calculated using age and years of residence in Germany. Length of residence in Germany is calculated using survey year and immigration year.

Gathering legal status information from the SOEP required the use of several variables from various questionnaires that asked respondents about their residence permits to varying degrees of detail. Since respondents were not asked for detailed legal status information in every survey year, in some cases legal status was imputed from the previous year or a more general indicator of permanent or temporary residency was utilized instead^5^. Responses were consolidated into a measure indicating four residence statuses: 1) legal protection or refugee status, 2) permanent residency, which also includes naturalized citizens, 3) temporary residency, 4) unknown or none, with 1% of respondents in the last category. Refugee samples were generally asked more detailed information about their current residence permit than the migration samples or the general sample. Detailed legal status information and information about imputed values are available in the Supplementary Table S3.

The final dataset includes 7,464 person-year observations and 4,247 unique person observations. Of this sample, descriptive statistics for person-year observations are reported in Table 1.

**Table 1:** Descriptive statistics of variables for full sample and immigrant groups.

	**(1)**	**(2)**	**(3)**	**(4)**
	**Full sample**	**EU**	**Third country**	**Refugee**
	**(% or mean)**	**(% or mean)**	**(% or mean)**	**(% or mean)**
**Receiving UB II**	41.71	15.68	24.73	60.50
**Employment level**				
Full Time	22.74	41.98	29.56	10.96
Part Time	20.89	24.91	27.88	16.36
Unemployed	56.38	33.11	42.56	72.68
**Years of residence in Germany**	7.82	10.25	12.38	4.96
**Age at immigration**	31.57	31.40	29.49	32.42
**Number of children**				
None	41.45	47.55	37.73	39.90
One	17.48	25.53	20.98	12.32
Multiple	41.06	26.92	41.29	47.78
**Single parent**	8.49	6.76	10.05	8.75
**Sex**				
Male	61.12	49.51	45.84	72.35
Female	38.88	50.49	54.16	27.65
**Age**	39.39	41.65	41.87	37.38
**Legal status**				
Asylum recipient	44.95	–	–	83.19
Permanent	43.13	95.13	68.90	8.21
Temporary	10.58	4.07	31.10	6.12
Unknown or none	1.34	–	–	2.48
**Education level (ISCED)**				
Primary	22.35	5.00	8.31	35.88
Lower secondary	18.81	14.85	18.43	20.85
Upper secondary	23.11	31.98	24.46	18.35
Post-secondary non-tertiary	4.93	8.92	6.17	2.55
Short-cycle tertiary	0.11	0.26	0.00	0.07
Bachelor's or equivalent	27.81	34.24	38.61	20.73
Master's or equivalent	0.56	0.88	1.47	0.07
Doctoral or equivalent	2.32	3.87	2.55	1.49
**Full-time employment (years)**	10.79	13.80	11.22	9.19
**Unemployment (years)**	1.72	1.05	1.71	2.04
**Language abilities**				
None (“gar nicht”)	0.95	0.48	0.57	1.61
Rather poor (“eher schlecht”)	12.29	8.05	8.00	14.83
Okay (“es geht”)	35.97	25.37	29.27	38.71
Good (“gut”)	35.62	38.58	33.62	34.33
Very good (“sehr gut”)	15.17	27.52	28.54	10.52
**Survey year**				
2013	6.97	11.60	15.42	1.61
2014	3.59	6.45	7.31	0.84
2015	9.35	18.57	17.49	1.91
2016	7.38	14.29	13.87	1.66
2017	12.81	17.74	14.34	9.87
2018	30.57	17.12	17.36	41.93
2019	29.33	14.23	14.21	42.18
**States**				
Schleswig-Holstein	3.68	1.55	3.42	4.81
Hamburg	2.63	2.63	3.62	2.26
Lower Saxony	7.98	6.55	8.31	8.55
Bremen	1.25	0.52	0.94	1.71
North Rhine-Westphalia	23.42	21.87	25.20	23.51
Hessen	9.74	8.97	11.39	9.50
Rhineland-Palatinate	5.71	6.76	5.97	5.11
Baden-Württemberg	13.17	14.80	13.34	12.32
Bavaria	15.66	21.92	14.21	13.19
Saarland	1.94	1.08	1.27	2.60
Berlin	4.84	6.76	4.09	4.19
Brandenburg	2.09	0.88	1.21	3.00
Mecklenburg-Vorpommern	0.99	0.52	0.34	1.46
Saxony	2.59	1.96	2.48	2.93
Saxony-Anhalt	1.90	0.88	2.08	2.33
Thuringia	2.41	2.37	2.14	2.53
**Region of country of origin** ^†^				
EU Old	8.68	33.21	–	0.10
EU New	17.12	65.55	–	0.17
Europe Other	0.88	1.24	2.08	0.27
Turkey	3.54	–	15.15	0.94
Ex-Yugoslavia	4.73	–	14.34	3.45
Former Soviet Union	8.43	–	35.19	2.58
Middle East/North Africa	40.49	–	8.71	71.71
North America	0.54	–	2.68	–
South America	1.10	–	5.36	0.05
Africa	6.15	–	4.49	9.72
Asia	8.17	–	11.13	11.01
Oceania	0.17	–	0.87	–
(N)	7,714	1,941	1,508	4,265

^*^p < 0.05, ^**^p < 0.01, and ^***^p < 0.001.

† Categorizations derived from the World Bank Databank: https://data.worldbank.org/.

### 3.3. Econometric model

I employ logit random effects models in the analysis, with the binary outcome indicating whether the respondent is receiving UB II in the given survey year. Random effects models were chosen over fixed effects models due to insufficient within-individual variation. As I am interested in between-persons variation, rather than within-person variation, random effects models were also a more appropriate fit. This structure allows for an examination of the differences between immigrant groups and immigrants with different legal statuses.

I first use stepwise models to examine predictors of welfare use among the three specified immigrant groups. Model 1, which only includes the immigrant group dummy variables, demonstrates the baseline difference between immigrant groups. Model 2 incorporates human capital factors including education, work experience, and German language skills. I also control for the time respondents have already spent unemployed. Survey year is also included to control for external economic conditions and any other time-specific effects. In Model 3, I add household composition, gender, and single parenthood in order to examine whether welfare use is largely a consequence of sociodemographic factors, as indicated by several previous studies (Riphahn, 1998; Castronova et al., 2001; Barrett and Maître, 2013; Bruckmeier and Wiemers, 2017). In Model 4 I add descriptors of respondents' migration experience, including legal status, age at immigration, and years of residence in Germany. To capture country effects, I also run a model with dummy variables for the largest country of origin to ensure a sufficient number of observations in each country group. Finally, I run separate models for each immigrant group with the country-of-origin dummies. Results of all models including these dummies are available in the Supplementary Table S6.

Random effects models typically do not employ both time-variant and time-invariant variables together. However, the panel data includes a combination of independent variables that generally do not change for each respondent over time (such as gender) and variables that may change over time (such as full-time employment experience). To address this challenge, within-between random effects models (Allison, 2009, 2014; Bell et al., 2019) allow for a combination of fixed effects estimates of the effects of time-variant variables with random effects-type estimates of the effects of time-invariant variables. To measure the effects of these time-varying variables, I obtain the standard deviation of the variable by subtracting the mean of observations from its observed value in each survey year (Schunck, 2013). As such, I can estimate the effect of a change in this variable by one standard deviation.

In order to determine which variables were time-varying and therefore appropriate for this within-between random effects model treatment, I first examined the percentage of each variable that changed over time for respondents. More detail about the percentage of individuals who experience change in any of the above variables is available in Table 2 below. As less than 5% of respondents indicated changes in education level, gender, number of children in the household, single parenthood, and legal status, these variables were treated as time-invariant. Age at immigration is also time-invariant as it is an event that occurred in the past. More than 10% indicated changes in their level of self-evaluated German language skills, full-time employment experience, and unemployment experience over the survey period. Years of residence in Germany also changes with each year of the survey period. These variables were thus treated as time-varying. Language skill is employed as a continuous variable in order to treat it as time-varying.

**Table 2:** Within variation of variables.

	**Within standard deviation**	**Percentage of person observations for whom value changes during survey period (*N* = 5,494)**
Unemployment benefits II receipt	0.217	10.52
Unemployment experience	0.257	12.23
Education level	0.054	0.82
Full-time employment experience	0.492	14.87
German language skills (spoken)	0.322	11.08
Gender	0	0
Number of children	0.178	4.55
Single parenthood	0.081	1.48
Legal status	0.177	2.82
Age at immigration	0	0
Years of residence in Germany	0.870	45.21

## 4. Results

### 4.1. Results of stepwise models

The results of the within-between random effects logistic regressions allow for an examination of the effects of group-level characteristics over the survey period. Marginal effects of stepwise models are reported in Table 3 and regression coefficients are reported in the Supplementary Table S5. At the baseline, third country immigrants are 7.4% more likely to use UB II than EU immigrants; refugees are 43.8% more likely to use UB II than EU immigrants. After accounting for all independent variables, this difference decreases: third country immigrants are 5.3% more likely to receive benefits than EU immigrants, and refugees are 30.6% more likely to receive benefits^6^. These results confirm hypothesis (H2a), that *EU immigrants exhibit the lowest rate of welfare use compared to refugees and third country immigrants*, as well as hypothesis (H2b), that there is an *additional refugee penalty predicting higher use of welfare benefits, even after accounting for human capital and sociodemographic factors*. Differences between the three immigrant groups remain significant even after controlling for country of origin, as demonstrated in the models that employ country-of-origin dummies (Supplementary Table S6).

**Table 3:** Likelihood of welfare receipt of immigrants in Germany, marginal effects, stepwise modeling with full sample.

	**Model 1**	**Model 2**	**Model 3**	**Model 4**
**Immigrant group (ref = EU immigrants)**				
Third country	0.074^***^ (0.017)	0.077^***^ (0.018)	0.058^**^ (0.018)	0.053^**^ (0.020)
Refugee	0.448^***^ (0.014)	0.399^***^ (0.018)	0.400^***^ (0.018)	0.306^***^ (0.028)
**Unemployment experience**	–	0.104^***^ (0.015)	0.110^***^ (0.015)	0.132^***^ (0.018)
**Education level (ref = low education)**				
Medium education	–	–0.071^***^ (0.016)	–0.057^***^ (0.015)	–0.059^***^ (0.015)
High education	–	–0.139^***^ (0.015)	–0.118^***^ (0.015)	–0.122^***^ (0.015)
**Other human capital**				
Full-time employment experience	–	–0.092^***^ (0.012)	–0.084^***^ (0.012)	–0.055^***^ (0.014)
German language abilities (spoken)	–	–0.017 (0.011)	–0.018 (0.011)	–0.014 (0.011)
**Sociodemographic factors**				
One child	–	–	0.006 (0.017)	–0.009 (0.017)
Multiple children	–	–	0.107^***^ (0.013)	0.084^***^ (0.013)
Female	–	–	0.067^***^ (0.013)	0.065^***^ (0.013)
Single parent	–	–	0.169^***^ (0.022)	0.181^***^ (0.022)
**Legal status (ref = temporary residency)**				
Recognized refugee	–	–	–	0.003 (0.029)
Permanent residency	–	–	–	–0.086^***^ (0.023)
**Migration-related factors**				
Age at immigration	–	–	–	0.007^***^ (0.001)
Years of residence in Germany	–	–	–	–0.024^*^ (0.010)

* *p* < 0.05,

** *p* < 0.01,

and

*** *p* < 0.001.

† indicates time-changing variables.

Several human capital factors remain significant across models. Not unexpectedly, education decreases likelihood of UB II receipt. Immigrants with a medium level of education (–0.059, *p* < 0.001), are less likely to receive UB II than those with a low level, and immigrants with a high level of education even less likely to receive UB II (–0.122, *p* < 0.001). In contrast to education, employment experience loses significance after accounting for sociodemographic characteristics, possibly because individuals lacking employment experience are engaged in childcare duties^7^.

Results of control variables related to sociodemographic characteristics are largely in line with the existing literature. Women (0.065, *p* < 0.001) and single parents (0.181, *p* < 0.001) display a higher likelihood of welfare receipt across immigrant groups. Having children in the household only becomes significant if there is more than one child (0.084, *p* < 0.001).

Finally, factors related to immigration significantly predict likelihood of welfare receipt; those who migrated to Germany at an older age are slightly more likely to receive benefits (0.007, *p* < 0.001), and those who have resided in Germany for a longer period of time are less likely to receive benefits (–0.024, *p* < space 0.05). Although legal status is not the focus of this paper, it is observed that legal status also predicts UB II receipt, even after controlling for immigrant group. Most notably, permanent residency or citizenship is related to a lower likelihood of welfare use (–0.086, *p* < 0.001). This finding corroborates previous evidence that permanent legal status or naturalization can potentially support immigrants' labor market success (Von Haaren-Giebel and Sandner, 2016; Gathmann and Keller, 2018; Riphahn and Saif, 2019). There are several possible mechanisms that may drive this relationship. Formal restrictions may limit temporary immigrants' access to certain jobs; employers may be more willing to hire immigrants who they know will stay in Germany; and immigrants may be incentivized to invest in host-country human capital once they know they will stay. That said, further research is needed to determine whether this result may be due to selection effects, where the most economically successful immigrants are also able to gain permanent residence or naturalization.

### 4.2. Results of interaction model

I also employ an additional model with an interaction term between immigrant group and education level. All independent variables from Model 4 are included as well. This model tests (H1) concerning returns to education by immigrant group.

**Figure 1:**
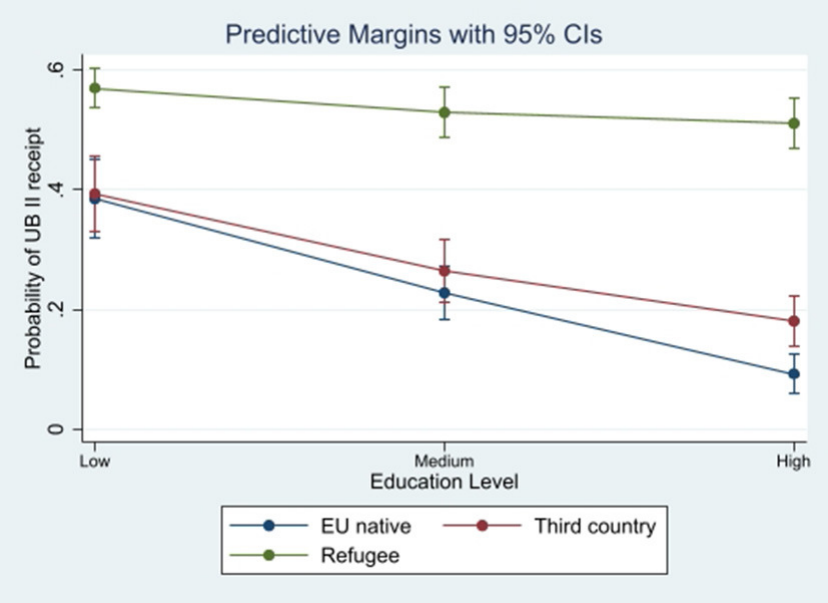
Predictive marginal effects of education level on probability of UB II receipt by immigrant group. Vertical lines mark 95% confidence intervals. Source: SOEP, own calculations.

Results are reported in Table 4 and indicate that education decreases the likelihood of welfare receipt for EU and third country immigrants. Compared to the baseline category of low education, coefficients for medium and high education are significant and negative for these two immigrant groups. The magnitude of these coefficients is similar as well, with a medium level of education reducing likelihood of welfare receipt by 13.9% for EU immigrants and 12.4% for third country immigrants. A high level of education reduces likelihood of welfare receipt by 24.3% for EU immigrants and 20.1% for third country immigrants. As demonstrated in Figure 1, the effect of education on likelihood of welfare receipt is therefore somewhat greater for EU immigrants than for third country immigrants.

**Table 4:** Likelihood of welfare receipt of immigrants in Germany, marginal effects of interaction term between education level and immigrant group.

**Interaction term: education level and immigrant group (ref = low education)**	
Medium education, EU immigrant	–0.139^***^ (0.031)
Medium education, third country	–0.124^***^ (0.036)
Medium education, refugee	–0.041 (0.021)
High education, EU immigrant	–0.243^***^ (0.030)
High education, third country	–0.201^***^ (0.034)
High education, refugee	–0.059^*^ (0.021)

* *p* < 0.05,

**p < 0.01, and

*** *p* < 0.001.

For refugees, a medium level of education does not significantly reduce the likelihood of welfare receipt compared to a low level of education, though this coefficient just barely lacks significance. A high level of education reduces likelihood of welfare receipt by 5.9%. The effect of a high level of education is therefore significantly smaller in magnitude for refugees compared to the other two immigrant groups.

These results only partially confirm hypothesis (H1a), that there is *a greater negative effect of educational attainment on welfare receipt for EU immigrants than for third country immigrants and refugees*. Rather, the effect of educational attainment on likelihood of welfare receipt is similar for EU and third country immigrants. Refugees diverge from the patterns displayed by other immigrant groups as the magnitude of the effect of education is much smaller for this group. Results remained significant even after the inclusion of the country-of-origin dummy variables as a robustness check.

## 5. Limitations

Several limitations of the study should be brought to attention. For one matter, many answers to questions asked in the SOEP are self-reported, which can lead to survey response bias (Stephan and McCarthy, 1958; Furnham, 1986; Lynn et al., 2012). For example, respondents were asked to rate their own German language abilities. Other answers may have been affected by misconceptions, as some respondents may have confused different types of benefits (Bruckmeier et al., 2021). Finally, recall bias (i.e., forgetting), confusion due to different benefits being claimed simultaneously, or social desirability bias due to stigma can all lead to underreporting of welfare receipt (Moffitt, 1983; Bound et al., 2001; Bruckmeier et al., 2014, 2021; Krafft et al., 2015).

A second limitation of the study is missing data. As education and training play a central role in my analysis, it would have been beneficial if the SOEP had included complete information about whether degrees acquired abroad were recognized in Germany. However, this information is unfortunately incomplete for most of the sample, and thus could not be included in the analysis. Among those for whom this data was available (approximately 40% of the sample), immigrants with recognized degrees were significantly less likely to receive UB II even after controlling for education level. Some highly educated refugees had degree recognition applications that were still in process at the time of the survey^8^. The SOEP also lacks information for the majority of the sample about whether immigrants obtained additional education or training after arriving in Germany. Finally, the study does not capture whether respondents have other sources of income or support when unemployed.

A third limitation of the study is potential panel attrition. It is possible that some immigrants return to their home countries during difficult times, thus forgoing German welfare state benefits. This may be particularly common among EU immigrants, who do not have to travel as far to go home (Zaiceva and Zimmermann, 2016). Return migration could therefore lead to attrition especially among EU immigrants and an underestimation of their need for unemployment benefits.

Although my analysis utilizes longitudinal data, it provides limited insights into the long-term labor market trajectories of many immigrants. Whereas, EU immigrants in the sample have resided in Germany for a median of 8 years and third country immigrants a median of 13 years, the refugees in the sample have resided in Germany for a median of 4 years. The SOEP is also an unbalanced panel with some variation in panel attrition in different samples, meaning that it does not provide a complete picture of each respondent's welfare receipt over the entire survey period (Siegers et al., 2020). For these reasons, continued research could paint a better long-term picture of the labor market trajectories of immigrant groups.

Finally, the categorization of immigrants into three broad groups—EU natives, third country immigrants, and refugees—is not completely unarbitrary. Quantitative analysis requires some generalization of heterogeneous groups; even immigrants from the same region are characterized by a variety of migration motives, social conditions, degrees of human capital transferability, and so on. However, they do share commonalities based on legal status, country of origin, and other aspects of the migration experience itself, such as migrating within the EU or as a refugee. These categorizations bear similarity to other papers (Wunder and Riphahn, 2014; Söhn, 2019). That said, in order to address this limitation, regressions with dummy variables for the largest countries of origin are available in the Supplementary Materials and do not affect the significance of the three broad categories (Supplementary Table S6).

## 6. Discussion and conclusions

Germany is now largely considered a “country of immigration,” with more than one in ten residents lacking German citizenship (Federal Statistical Office, 2022). As its immigrant population continues to grow, the beneficiaries of the German welfare state continue to diversify. Immigrants arrive with educational qualifications from many different countries, a diversity of professional experience and training, and varied language skills. Policymakers will need to ask themselves how they can support a thriving labor market in a country of immigration. With such challenges in mind, my study aims to investigate the potential influence of human capital on UB II receipt for immigrants from various countries of origin and with different migration pathways. I investigate whether different immigrant groups exhibit different likelihoods of welfare use, the role of human capital in predicting welfare use for each group, and whether differences remain after accounting for human capital. My results have several implications for labor market integration and welfare state policies and point to several avenues for further investigation. I find evidence for two possible mechanisms: 1) the devaluation of non-Western human capital, or 2) what I call the refugee penalty.

Overall, EU immigrants are the least likely to receive welfare benefits. The addition of human capital, sociodemographic, and migration-related factors across stepwise models decreases differences between immigrant groups, supporting previous research by Wunder and Riphahn, who largely attribute disparities in welfare receipt to differences in education and family structure. However, like Wunder and Riphahn, I find that some disparities between immigrant groups remain even after accounting for all these factors, with a higher likelihood of welfare use among third country immigrants, and even higher among refugees. These disparities merit further investigation and indicate that research on the welfare use of immigrants can benefit from separating refugees from other third country immigrants.

Disparities in welfare receipt may tentatively indicate that EU immigrants enjoy greater returns to human capital, and consequently lower welfare use, than those from third countries. Education from abroad is valued according to both the perceived quality of the education system and its comparability to the German system (Basilio et al., 2017). Degrees and qualifications from Western countries possess greater signaling value, easing the process of finding employment (Friedberg, 2000; Lancee and Bol, 2017). Such differences in the valuation of human capital by country of origin are symptomatic of Eurocentrism and hierarchizing between Western and non-Western countries (Said, 1978). Labor market policies to support recognition of skills and qualifications that fall outside the usual structure and scope of the German system can help rectify such inequalities. Policy measures can help ensure that immigrants can enter the labor market more quickly by making use of education and training obtained in the country of origin.

However, counter to previous research, EU and other third country immigrants are relatively similar in terms of how education impacts their likelihood of welfare receipt. It is refugees whose returns to education differ significantly from those of other immigrants. Furthermore, refugees remain the most likely to receive UB II in all econometric models, regardless of education level, and while other third country immigrants are more likely to receive UB II than EU immigrants, refugees' likelihood of welfare receipt far exceeds that of the other immigrant groups. These results may indicate that refugees' human capital is particularly undervalued in Germany in formal and informal arenas.

Rather than indicating a devaluation of non-Western human capital, results could also point to a “refugee penalty.” There are several possible mechanisms that may impact the labor market outcomes of refugees and contribute to this penalty. Discrimination and mental health challenges could hinder labor market integration especially among this group (Ruiz and Vargas-Silva, 2018; Khan-Gökkaya and Mösko, 2021; Kloubert and Hoggan, 2021). Conversely, more recently arrived and highly educated refugees might have been provided with additional assistance accessing social benefits or might have been able to navigate the welfare bureaucracy more easily due to their education and skills. Instead of a labor market disadvantage, this possibility would mean that refugees have some advantage over other immigrants in terms of access to social benefits.

In particular, the process of entering Germany as a refugee and obtaining asylum can have long-term labor market penalties. Söhn (2017) and Schuss (2019) find that asylum seekers face employment disadvantages years after moving to Germany, even after accounting for education level and length of residency. Schuss attributes refugees' labor market disadvantages to their residency status upon arrival. Protracted asylum processing times, extended time spans between arrival and permission to work, and insecurity about the future all may contribute to delayed employment even long after arrival in Germany (Schuss, 2020; Kosyakova and Brenzel, 2020). Such setbacks in labor market entry have been demonstrated to leave lasting impacts such as long-term income disadvantages (Söhn et al., 2017; Schuss, 2020). By preventing labor market entry, delays in asylum case processing may direct refugees toward a path of benefit dependency.

Schuss' findings also highlight the potential role of legal status in labor market outcomes. Although the aim of this study was not to explore the role of legal status in welfare use, some interesting results came to light when legal status was used as a control variable. Permanent residents and citizens are significantly less likely to receive welfare benefits than temporary residents or those with a temporary refugee status. This finding is in line with previous research on the role of naturalization for labor market integration: several studies in Germany have indicated that naturalization can lead to positive labor market outcomes (Von Haaren-Giebel and Sandner, 2016; Gathmann and Keller, 2018; Riphahn and Saif, 2019; Brunow and Jost, 2021). However, the labor market implications of permanent residency have received little scholarly attention. Jutvik and Robinson (2020), in Sweden, find that refugees granted permanent residency demonstrate poorer labor market outcomes than temporary residents. At the same time, they also find that permanent residency can incentivize investment in country-specific skills such as language and vocational training (Gathmann and Keller, 2018; Jutvik and Robinson, 2020). Dustmann (1993) theorizes that immigrants are more likely to invest in host-country human capital if they can expect to remain in the destination country, potentially reducing the long-term risk of welfare receipt (Dustmann, 1993; Duleep and Regets, 1999; Cortes, 2004). Naturalization or permanent residency can also indicate to employers that immigrants intend to remain permanently in Germany, thus making them more attractive hires (Von Haaren-Giebel and Sandner, 2016; Riphahn and Saif, 2019). Finally, while temporary residents receive only conditional labor market access, permanent residents and recognized refugees are generally able to take any job (Etzold, 2017; Gathmann and Keller, 2018; Brunow and Jost, 2021).

Considering the empirical evidence, immigration reforms can support immigrants' and particularly refugees' labor market outcomes. While policy goals regarding the legal status of immigrants may remain divided, labor market integration is generally in the interest of both immigrants and host country welfare states. Reduced asylum application processing times and quicker access to the labor market can help support the long-term employment outcomes of refugees (Söhn et al., 2017; Schuss, 2020; Kosyakova and Brenzel, 2020). Receiving an asylum decision increases the transition rate to the first job and investment in German language acquisition (Kosyakova and Brenzel, 2020). Furthermore, my results support the notion that a secure legal status and rapid access to the labor market can contribute to reducing labor market disadvantages (Bauder, 2008). The 2016 Integration Act determined that recognized refugees would be granted permanent residency after 5 years instead of the previous threshold of 3 years (Hanewinkel and Oltmer, 2018). My results as well as those of other papers suggest that such restrictions on permanent residency may hinder rather than facilitate labor market integration. However, further research is needed to determine whether my results are driven by selection effects: possibly, those who succeed in the labor market are more likely to be granted permanent residency. As Gathmann and Keller (2018) and Riphahn and Saif (2019) both find gender differences in the effects of naturalization on employment outcomes, further research should also take this into account.

This paper focuses on the role of human capital in predicting likelihood of welfare receipt. By indicating differences in returns to human capital by immigrant group, results also demonstrate that immigrants' labor market outcomes are influenced by much more than their education and work experience. For one matter, I provide evidence that immigrants' livelihoods are shaped by the legal contexts within which they operate (Sainsbury, 2012). Policymakers concerned with immigrants' labor market integration therefore cannot ignore the promises of a future in Germany that a secure legal status can provide. In addition, labor market trajectories may be affected by the replication of long-standing systemic inequalities that place value on human capital from some countries above human capital from others. If the goal of integration measures is to maximize immigrants' labor market outcomes and minimize the need for welfare benefits, measures to ease the transferability of origin-country human capital can support this goal. Policy changes such as recognition of foreign degrees, facilitation of labor market entry for those with foreign education, and validation of informal work experience would require a revision of what are considered acceptable and desirable qualifications in the German labor market. Such measures would reflect new conceptions of immigrant integration as a two-way process, whereby both newcomer minorities and the native majority are both expected to adapt to each other (Hellgren, 2015; Klarenbeek, 2021). Finally, the results of this analysis indicate that refugees still demonstrate higher levels of welfare use even after accounting for human capital, sociodemographic factors, and time in the destination country. Further research should explore potential explanations for this gap, including delays in labor market entry, devaluation of human capital, discrimination, or mental health challenges. Continued study of the refugee penalty should help policymakers ensure that immigrants, regardless of nationality, are able to enter the labor market successfully and use the human capital skills they already possess.

## Data availability statement

The data analyzed in this study is subject to the following licenses/restrictions: Data are available from the German Socio-economic Panel Study (SOEP) due to third party restrictions (for requests, please contact soepmail@diw.de). The scientific use file of the SOEP with anonymous microdata is made available free of charge to universities and research institutes for research and teaching purposes. The direct use of SOEP data is subject to the strict provisions of German data protection law. Therefore, signing a data distribution contract is a precondition for working with SOEP. Requests to access these datasets should be directed to http://www.diw.de/soepforms, or contact the SOEPhotline at either soepmail@diw.de or +49-30-89789-292.

## Author contributions

EF confirms sole responsibility for the following: study conception and design, data organization, analysis and interpretation of results, and manuscript preparation.

## Funding

This study was supported by the German Federal Ministry of Labour and Social Affairs (BMAS), Grant No. FIS.00.00066.19 and the Deutsche Forschungsgemeinschaft (DFG, German Research Foundation) - 390285477/GRK 2458.

## Conflict of interest

The author declares that the research was conducted in the absence of any commercial or financial relationships that could be construed as a potential conflict of interest.

## Publisher's note

All claims expressed in this article are solely those of the authors and do not necessarily represent those of their affiliated organizations, or those of the publisher, the editors and the reviewers. Any product that may be evaluated in this article, or claim that may be made by its manufacturer, is not guaranteed or endorsed by the publisher.

## Footnotes

1 While UB II recipients are required to be between the ages of 15 and 65, not 18 and 65, there are no immigrant household heads in the SOEP under the age of 18.

2 Benefit regulations for EU citizens do not require that EU immigrants engage in full-time work as opposed to part-time work, but as the information available in the SOEP is based on full-time work experience, I rely on this variable as a measure of work experience for sample specification purposes.

3 Age is not employed as a separate control variable because it is collinear with age at arrival.

4 Those at the lowest ISCED level (0) are still in secondary school; those at ISCED level 1 have completed primary education, while those at level 8 have completed a doctoral degree.

5 These more general indicators were utilized for approximately 40% of observations. Reported legal statuses with very few observations were consolidated with others; for example, 16 family reunification observations were combined with the more general subsidiary protection status, which applies equivalent regulations, and those with “applied for new residence permit” were consolidated with the “unknown” category.

6 I also ran a model that included employment level, in order to control for whether some respondents may be engaged in work that does not generate sufficient income to rise above the poverty line. This model rendered full-time employment experience and gender insignificant.

7 I also ran a set of models, not reported here, that treat German language skills as a discrete variable with the above-mentioned five-point scale, instead of as a continuous variable. In those models, very good language skills significantly decrease likelihood of welfare receipt only before accounting for immigration-related factors, potentially indicating that length of residency in the destination country supports language acquisition.

8 Degree recognition information is available for 85% of highly educated refugees in the sample. Among those for whom information is available, 56% of observations did not apply for recognition of their degrees. In total, information on the outcome of the degree recognition process is therefore available for a total of 425 observations. Of those observations, 48% reported that their degrees had been recognized, 1% that they had been partially recognized, 9% that they had been rejected, and 42% were still in progress.
