# Supplementary material for: Determinants of welfare benefit use of immigrant groups - longitudinal evidence from Germany
Source: Front Sociol. 2022 Nov 4;7:839352. doi: 10.3389/fsoc.2022.839352 (PMC9672685; doi:10.3389/fsoc.2022.839352)
Supplement: Supplementary file 1 [file Data_Sheet_1.pdf]

Table S1. Rights to UB II by residence permit.

Residence permit	Rights to Unemployment Benefits II
Asylum seeker	Not eligible; receive asylum seekers' benefits instead.
Stay of deportation	Not eligible; receive asylum seekers' benefits instead.
Recognized refugees	Eligible
EU citizens	Yes, after 1 year of working or 5 years of residence.
Third-country migrants without permanent residence	Eligible, but could jeopardize applications for naturalization & permanent residency. Must have either resided in Germany for at least 5 years, previously received UB I, or worked for at least 3 of the last 6 months and ineligible for UB I.
Third-country migrants with permanent residence	Eligible, but could jeopardize applications for naturalization.

Detailed information including all exceptions to regulations available at Informationsverbund Asyl & Migration, 2022.

Table S2. Information about cases excluded from the original sample of all first-generation immigrants in the SOEP, 2013-2019 survey years.

	Person observations left after restriction	Person-year observations left after restriction
**Stages of sample selection**		
Original sample	18,621	54,661
**Keep if:**		
Household head	11,111	32,921
aged 18-65 at time of interview	10,678	30,763
not on parental leave	10,577	29,632
not receiving old age, disability, or civil service pension	8,652	23,196
EU citizens: legal eligibility rules*	8,637	23,145
non-missing legal status variable	6,562	20,998
asylum applicants: legal eligibility rules**	5,994	13,663
analytical sample	5,494	10,355

* EU citizens who have lived less than 5 years in Germany and have less than one year of part- or full-time work experience are excluded.

** Asylum seekers without protection status are excluded.

Table S3. Detailed legal status information.

**Broad category**	**Detailed category**	**EU native**	**Third country**	Refugee
**Recognized refugee**	Recognized refugee (*Asylberechtigt*)	–	–	238
	Recognized refugee (*Flüchtlingseigenschaft*)	–	–	2,352
	Recognized refugee (*subsidiärer Schutz*)	–	–	929
	Recognized refugee (*humanitäre Aufnahme*)	–	–	225
**Permanent residency**	EU citizenship	1,036	275	51
	Permanent residency (*Niederlassungserlaubnis*)	85	476	202
	Permanent residency (no further information)	1,877	1,026	286
**Temporary residency**	EU Blue Card	21	10	6
	Temporary residency (no further information)	99	676	371
**Unknown**	Other residence permit	–	–	114

Detailed legal status information was asked for 6,020 out of the full analytical sample of 10,355 person-year observations. Information about EU citizenship was then imputed for individuals who migrated from EU countries. For some person-year observations, a more general legal status question was asked (permanent or temporary residency), and so this information was used for any subsequently missing observations. Finally, leftover missing values were filled in by imputing the previous year's legal status, as some respondents, particularly in SOEP samples M1 and M2, were not asked about their legal status on a yearly basis.

For the general legal status variable used in the regressions, all recognized refugee categories were consolidated. EU citizenship and all permanent residency categories were consolidated into one permanent residency category. EU Blue Card and temporary residency were consolidated into one temporary residency category.

To construct the broad legal status variable from the detailed legal status variable, some imputation was used: when available, detailed legal status information was imputed from the previous year. When unavailable, general legal status information (temporary or permanent residency) was imputed from the same year.

Table S4. Operationalization of model components.

Variable name	Variable definition	SOEP variables used	Variable type; unit, measure, range
**UB II**	Indicates whether respondent's household is receiving Unemployment Benefits II.	hlc0064_v2, plc0132_v2, plc0132_v3	Binary (0 = not receiving, 1 = receiving)
**Immigrant group**	Indicates whether the respondent migrated from an EU country, a third country, or as a refugee.	corigin, arefback	Categorical (1 = EU native, 2 = third country, 3 = refugee)
**Unemployment experience**	Indicates length of time for which respondent has been unemployed in his/her lifetime.	pgexpue	Continuous; years
**Education level**	Respondent's level of education using the International Standard Classification of Education (ISCED) scale, simplified into three categories.	pgisced11	Categorical (1 = less than upper secondary, 2 = upper secondary/post-secondary non-tertiary/short-cycle tertiary, 3 = Bachelor's degree and above)
**Qualifications from abroad**	Indicates whether respondent's highest educational qualification was acquired abroad or in Germany (used as sampling restriction).	pgpsbila, pgpbbila, lb0186_v1, lb0186_v2, plm0625	Binary (0 = in Germany, 1 = abroad)
**Full-time employment experience**	Indicates length of time for which respondent has held full-time employment in his/her lifetime.	pgexpft	Continuous; years
**Language abilities**	Spoken German language abilities, self-evaluated.	plj0071, lb1191, bepm_p_23801	Categorical; Likert scale 1-5 (1 = none, 2 = rather poor, 3 = okay, 4 = good, 5 = very good)
**Number of children**	Indicates whether a household contains no children, one child, or multiple children.	d11107	Categorical (0 = no children, 1 = 1 child, 2 = multiple children)
**Single parenthood**	Respondent is a single household head of a household with children. Created using indicators of whether respondent is partnered and their number of children.	partner, d11107	Binary (0 = not single parent, 1 = single parent)
**Gender**	Indicates if respondent is female.	sex	Binary (0 = male, 1 = female)
**Legal status**	Respondent's legal residence status in Germany at time of survey, with simplified categories for analysis.	plj0680_v1, plj0680_v2, plj0722, lb1240_v1, lb1240_v2, lb0021, pgstatus_refu, biimgrp, biresper	Categorical (1 = asylum applicant/stay of deportation, 2 = recognized refugee, 3 = permanent residency, 4 = temporary residency, 5 = unknown
**Age at immigration**	Calculated by subtracting years of residence in Germany from age at time of survey.	d11101, syear, immiyear	Continuous; years
**Years of residence in Germany**	Years of residence in Germany since migration. Created by subtracting immigration year from survey year.	syear, immiyear	Continuous; years
**Employment level**	Indicates whether respondent is employed full-time, employed part-time, or not working.	e11103	Categorical (1 = full-time, 2 = part-time, 3 = not working)

Table S5. Likelihood of welfare receipt of immigrants in Germany, regression outputs.

	Model 1	Model 2	Model 3	Model 4
**Immigrant group (ref = EU immigrants)**				
Third country	1.022***	0.936***	0.716**	0.590**
	(4.45)	(4.22)	(3.27)	(2.62)
Refugee	4.702***	3.922***	3.948***	2.974***
	(20.62)	(15.87)	(15.78)	(9.88)
**Education level (ref = low)**				
Medium education	--	-0.713***	-0.583***	-0.601***
	--	(-4.51)	(-3.74)	(-3.94)
High education	--	-1.435***	-1.225***	-1.262***
	--	(-8.73)	(-7.63)	(-8.02)
**Unemployment experience**	--	1.097***	1.173***	1.411***
	--	(6.84)	(7.28)	(7.66)
**Other human capital**				
Full-time employment experience	--	-0.966***	-0.891***	-0.584***
	--	(-7.28)	(-6.76)	(-3.77)
German language skills (spoken)	--	-0.180	-0.186	-0.150
	--	(-1.49)	(-1.54)	(-1.23)
**Sociodemographic factors**				
One child	--	--	0.0655	-0.0908
	--	--	(0.36)	(-0.50)
Multiple children	--	--	1.112***	0.868***
	--	--	(7.84)	(6.30)
Single parent	--	--	1.757***	1.877***
	--	--	(7.33)	(7.94)
Female	--	--	0.714***	0.693***
	--	--	(4.97)	(4.95)
**Legal status (ref = temporary residency)**				
Recognized refugee	--	--	--	0.0280
	--	--	--	(0.10)
Permanent residency	--	--	--	-0.871***
	--	--	--	(-3.84)
**Migration-related factors**				
Age at immigration	--	--	--	0.0755***
	--	--	--	(10.73)
Years of residence in Germany	--	--	--	-0.253*
	--	--	--	(-2.48)
N	7,464	7,464	7,464	7,464

t statistics in parentheses

* $p < 0.05$, ** $p < 0.01$, *** $p < 0.001$

Table S6. Marginal effects of models by immigrant group including largest countries of origin.

	(1)<br>EU immigrants	(2)<br>Third country	(3)<br>Refugee	(4)<br>Full sample
**Immigrant group (ref = EU immigrants)**				
Third country	--	--	--	0.095* (0.037)
Refugee	--	--	--	0.326*** (0.043)
**Unemployment experience**	0.079*** (0.019)	0.069*** (0.020)	0.191*** (0.036)	0.133*** (0.018)
**Education level (ref = low education)**				
Medium education	-0.129*** (0.030)	-0.134*** (0.038)	-0.046* (0.021)	-0.064*** (0.015)
High education	-0.223*** (0.030)	-0.199*** (0.038)	-0.064** (0.021)	-0.125*** (0.015)
**Other human capital**				
Full-time employment experience	-0.008 (0.014)	-0.066** (0.020)	-0.160*** (0.041)	-0.054*** (0.015)
German language abilities (spoken)	-0.015 (0.016)	-0.023 (0.020)	-0.010 (0.018)	-0.014 (0.011)
**Demographic factors**				
One child	-0.017 (0.021)	-0.054 (0.029)	0.033 (0.029)	-0.013 (0.017)
Multiple children	0.027 (0.023)	0.022 (0.028)	0.123*** (0.019)	0.076*** (0.013)
Female	0.076*** (0.020)	0.003 (0.025)	0.090*** (0.019)	0.066*** (0.013)
Single parent	0.259*** (0.051)	0.318*** (0.056)	0.089** (0.030)	0.182*** (0.022)
**Legal status (ref = temporary residency)**				
Recognized refugee	---	---	-0.061 (0.041)	-0.031 (0.029)
Permanent residency	-0.021 (0.039)	-0.061*** (0.025)	-0.147** (0.046)	-0.083*** (0.022)
**Migration-related factors**				
Age at immigration	0.004** (0.001)	0.007*** (0.002)	0.007*** (0.001)	0.007*** (0.001)
Years of residence in Germany	-0.010 (0.010)	-0.020 (0.013)	-0.020 (0.021)	-0.021* (0.010)
**Country of origin group (ref = other)**				
Greece	0.055 (0.046)	--	--	0.098 (0.064)
Italy	0.056 (0.039)	--	--	0.105 (0.054)
Spain	-0.039 (0.042)	--	--	-0.100 (0.075)
Romania	-0.005 (0.028)	--	--	0.010 (0.047)
Poland	0.021 (0.028)	--	--	0.072 (0.044)
Hungary	-0.062 (0.043)	--	--	-0.107 (0.085)
Bulgaria	0.150** (0.049)	--	--	0.216*** (0.054)

Turkey	--	0.016 (0.037)	--	0.039 (0.041)
Russia	--	0.005 (0.056)	--	-0.018 (0.046)
Kazakhstan	--	0.055 (0.056)	--	0.054 (0.058)
Ukraine	--	0.064 (0.054)	--	0.076
Kosovo/Albania	--	-0.105* (0.044)	-0.230** (0.078)	-0.149*** (0.042)
Iran	--	--	0.165** (0.055)	0.186*** (0.055)
Syria	--	--	0.058 (0.032)	0.071** (0.028)
Afghanistan	--	--	0.099** (0.040)	0.101** (0.037)
Iraq	--	--	0.097** (0.037)	0.097** (0.034)
Eritrea	--	--	-0.022 (0.044)	-0.019 (0.036)
N	1,939	1,492	4,033	7,464

* $p < 0.05$, ** $p < 0.01$, *** $p < 0.001$

† indicates time-changing variables
